# Glyph norming: Human and computational measurements of shape angularity in writing systems

**DOI:** 10.3758/s13428-025-02682-7

**Published:** 2025-05-14

**Authors:** Alexander Porto, Nikolai Huckle, Alexander Basalyga, Julio Santiago, Alexander Kranjec

**Affiliations:** 1 Department of Psychology, Duquesne University, 600 Forbes Avenue, Pittsburgh, PA 15219 USA; 2 Independent Researcher, Pittsburgh, PA 15282 USA; 3 Mind, Brain, and Behavior Research Center, University of Granada, Granada, Spain

**Keywords:** Writing systems, Stimuli development, Computational shape analysis, Cognitive linguistics

## Abstract

Writing systems are an underused source of stimuli for behavioral and computational experiments in cognitive psychology, psycholinguistics, and anthropology, despite being ecologically relevant and systematically different in shape, structure, and orientation. One possible reason that glyphs of writing systems are not commonly used in behavioral research concerns their profound complexity. However, recent developments in computer vision (i.e., geometric shape analysis) offer tools to automatically assess their visual dimensions. The current work describes an open-access database of 3,208 glyphs from diverse writing systems that have been normed by computational analyses in terms of shape angularity using an array of measurements. We further validate these norms by obtaining human judgments of angularity for a subset of 400 glyphs and show that they correlate highly with computational measures, in particular with first-order entropy of edge orientation. Additionally, we provide methods for standardized glyph generation based on Unicode ranges, a straightforward example of computational shape analysis, and a demonstration of automated transliteration of glyphs from Unicode strings using a pre-existing Python library. These procedures should facilitate the characterization of angularity of new glyphs and any other kind of visual shape by independent researchers. The present work will be helpful to scientists working across different topics in the various cognitive science subdisciplines.

**Supplementary information:**

The online version contains supplementary material available at 10.3758/s13428-025-02682-7.

## Introduction

Writing systems are an underused source of stimuli for behavioral and computational experiments in cognitive psychology, psycholinguistics, and anthropology, despite being ecologically relevant, potentially multimodal (i.e., interaction of speech and orthography via handwriting), and systematically different in shape, structure, and orientation. Moreover, writing systems contribute to a rich evolutionary story about human behavior and provide insight into cultural transmission (Miton & Morin, 2021; Morin, 2022).

One reason that glyphs of writing systems are not commonly used in behavioral research is that they are profoundly complex (Miton & Morin, 2021). For example, there is ostensibly only minimal overlap between, say, abjad, Ethiopic, and alphabetic systems, and even within a single system, say, alphabetic, there are substantial differences in shape, pronunciation, and complexity. For example, compare the Latin alphabet with Cyrillic, or consider how, say, German writing includes glyphs with umlauts, whereas English does not, despite both being built out of the Latin script. Such differences raise important questions: What parts of writing systems should we use for behavioral tasks? How do researchers avoid confounding aspects of writing (say, exposure to multiple writing systems, multilingualism, etc.)? What features of a writing system could be isolated for behavioral studies (e.g., shape, sound, orientation) and why?

Along different lines, developments in computer vision, geometric shape analysis, and similar computational methods have focused on the complexity of writing systems. For example, human handwriting can be used to train machine learning models for automated optimal character recognition and visual clustering algorithms (Braun et al., 2013; Kelly et al., 2020; Morin, 2018, 2022; Watier, 2024). Or, computer vision can leverage a robust computational battery to identify changing complexity features in the shape of writing systems over time (Miton & Morin, 2021). So, on the one hand, the complexity of writing hinders behavioral research, and on the other hand, the same complexity makes for exciting advancements in computational measures of language evolution.

The objective of this work is to establish an open-access database of glyphs from diverse writing systems that have been normed by computational analysis and human agents regarding the angularity of their shape. In particular, we present a set of 3,208 glyphs normed for shape angularity using an array of measurements. Additionally, we provide simple means for standardized glyph generation based on Unicode ranges, a straightforward example of computational shape analysis, and a demonstration of automated transliteration of glyphs from Unicode strings using a pre-existing Python library.

The primary rationale for this work was to develop a normed stimuli set for ongoing research that investigates the sound symbolic properties of real-world writing and speech. In particular, out of the many physical ways that glyphs could be categorized, we chose to norm for angularity. This is because such shape feature is well established as a dimension that motivates some forms of sound symbolism (i.e., the *kiki-bouba* effect). However, the present stimuli set is relevant to scientists working across different topics in the various cognitive science disciplines. For example, researchers working between empirical pedagogy and cognitive neuroscience could use the stimuli in reading and writing tasks; cognitive anthropologists could combine shape features of writing systems with other evolutionary data to better contextualize the development of writing; or experimental psychologists could replicate the computational shape feature detection to develop different stimuli sets for studies on attention and memory.

## Glyph selection and stimuli design

The stimuli set comprises 3,208 glyphs, representative of the world’s script families following Daniels and Bright’s five-way typology of the historical development of writing (Daniels, 2017; Daniels & Bright, 2007). The basic properties of each writing system are as follows: (1) Abjad (e.g., Arabic and Hebrew): Each glyph stands for a consonant; vowels are depicted through so-called vowel points. (2) Abugida (e.g., Thai, Tigrinya, Khmer, and Inuktitut): Each glyph stands for a consonant accompanied by a particular vowel, and other vowels (or none) are indicated by consistent additions to consonant symbols. (3) Alphabet (e.g., Georgian, Latin languages, Turkish): Each glyph stands for either a vowel or consonant. (4) Featural (e.g., Korean): Each glyph conveys phonological features of the represented phoneme. (5) Logosyllabary (e.g., Mandarin, Japanese Kanji): Each glyph stands for a monosyllabic morpheme and can be used to convey both sound and meaning. (6) Syllabary (e.g., Japanese Hiragana, Ryukyuan, and Palauan): Each glyph stands for a syllable. It is important to note that there is a substantial difference between systems that write speech (e.g., alphabets and syllabaries) and systems that write meaning (logographic systems). We refer interested readers to Daniels and Bright (2007) for further anthropological, historical, and linguistic detail.

The image files for 3,208 glyphs were produced in Python from Unicode ranges (see <glyph_gen.py> in the Supplementary Materials for code).^1^ The scripts include Latin, Cyrillic, Greek, Georgian, Hebrew, Arabic, Kanji, Cherokee, Yi, Hiragana, Katakana, Hangul, Thai, Ge’ez, and Devnagari. Glyphs were uniformly rendered in black and placed on transparent background, using Noto typefaces for each writing system. Each glyph was rendered in 500 × 500 px. Glyph generation was based on the methods used by Miton and Morin (2021).

For stimuli norming with human participants, we randomly sampled a set of 400 glyphs. It is important to note that the distribution of the writing systems in the human norming phase is unbalanced. There are two reasons for this: (1) we chose to exclude writing systems that participants were likely to be familiar with, and (2) we used random sampling for glyph selection from the entire set of generated glyphs, which included many more logograms than glyphs from syllabaries and alphabetic systems.

## Phase 1: Computational shape analysis and automated transliteration

### Overview

We sought to provide a mathematical measure of the degree of angularity (from round to angular) of the included glyphs.

### Methods

Following the computational approaches for feature detection of Redies et al. (2017) and Watier (2024), we performed analyses for the following metrics: *prominent peaks in S*(*θ*), *entropy of edge orientation*, *chord-to-point distance accumulation* (*CPDA*), and *scalar q* (described below). As our work replicates Watier’s (2024) method, we only briefly comment on the computational details of the selected metrics. We encourage interested readers to refer to the original work for more elaborate mathematical detail. The MATLAB scripts used in this study can be found in the Supplementary Materials. Readers interested in using the scripts should open all of the files into a working directory; the script titled <calculate_all_metrics.m> (which iterates over all of the scripts to calculate each metric) contains a list of dependencies.^2^ Interested readers also must specify the pixel dimensions of the image files in lines 22–23 in the <edge_entropy.m> script. Apart from installing dependencies and setting pixel parameters, the scripts are designed to run as is.

Each metric provides different measurements of the degree of angularity for a single image. *Prominent peaks in S*(*θ*) measures how energy in the magnitude spectrum of a set of pixels is distributed across orientations. Peaks in S(θ) indicate a local maximum of magnitude at specific orientations. As the number of peaks increases, there are greater concentrations of magnitude at a specific orientation, which is characteristic of angular shapes. In other words, given that “magnitude is concentrated along specific orientations in angular shapes” (Watier, 2024, p. 3), angular images will yield greater prominent peaks in S(θ).

*Entropy of edge orientation *(*first- and second-order*) measures the uniformity of the distribution of edges (i.e., lines which form the outline) of an image across orientations, plotted along a histogram. Images with greater uniformity of distribution of edge orientations are more curvilinear than angular. In *first-order entropy*, the edge distribution summarizes the orientations of all edges in a particular image; in *second-order entropy*, the edge distribution summarizes differences in orientation among nearby pairs of edges.

*CPDA* measures the Euclidean distance between a contour and a straight line. Curvature and Euclidean distance increase together. Suppose one draws a straight line through (1) the center of a triangle and (2) the center of a semioval with minimal convexity; in (1), the contoured line segments will have a greater distance from the straight line than (2), as the contours of the semioval will be much closer to the line than the triangle. Images with more angular features are indicated by greater CPDA.

*Scalar q* measures the magnitude of bends in a curve, relative to unit tangent vectors. As mean scalar q values increase, there are greater changes in the direction of the image gradient along a contour. Thus, the angularity of an image is detected by greater rates of scalar q.

After computing these metrics, we performed automated transliteration on the entire glyph set using AnyAscii (https://github.com/anyascii/anyascii).

## Phase 2: Human norming

### Overview

The aim of Phase 2 was to obtain human-generated norms for a subset of 400 glyphs from the original set of 3,208 in order to validate the computational measures.

### Methods

Participants (*n* = 73) were recruited from a pool of undergraduate and graduate students at a private university in North America. Demographic information was not collected. Informed consent was obtained from all participants included in the study.

### Procedure

An online experiment was built on lab.js and delivered via the open-lab.online server (Henninger et al., 2022; Shevchenko, 2022). Participants dichotomously classified the shape of a set of 400 glyphs in a forced-choice paradigm. Participants were shown a single glyph and were asked to classify it as round or angular. Every participant saw the same set of glyphs in a different random order.

## Results

First, we ran a baseline test analysis using the MATLAB scripts for the above metrics provided in Watier (2024) on the original image files from Bar and Neta (2007). Our test analysis generated identical results to the original study, which indicated that the scripts were working as intended. We then applied the scripts to the glyph corpus. The results can be found in the Glyph_Data.xlsx file.

The computational metrics discussed above are included as the following variables in the data: <peaks_s_theta> stands for prominent peaks in S(θ); <first_order> stands for first-order edge entropy; <second_order> stands for second-order edge entropy; <CPDA_maxima> stands for chord-to-point distance accumulation; and <scalar_q_maxima> stands for scalar q.

**Fig. 1:**
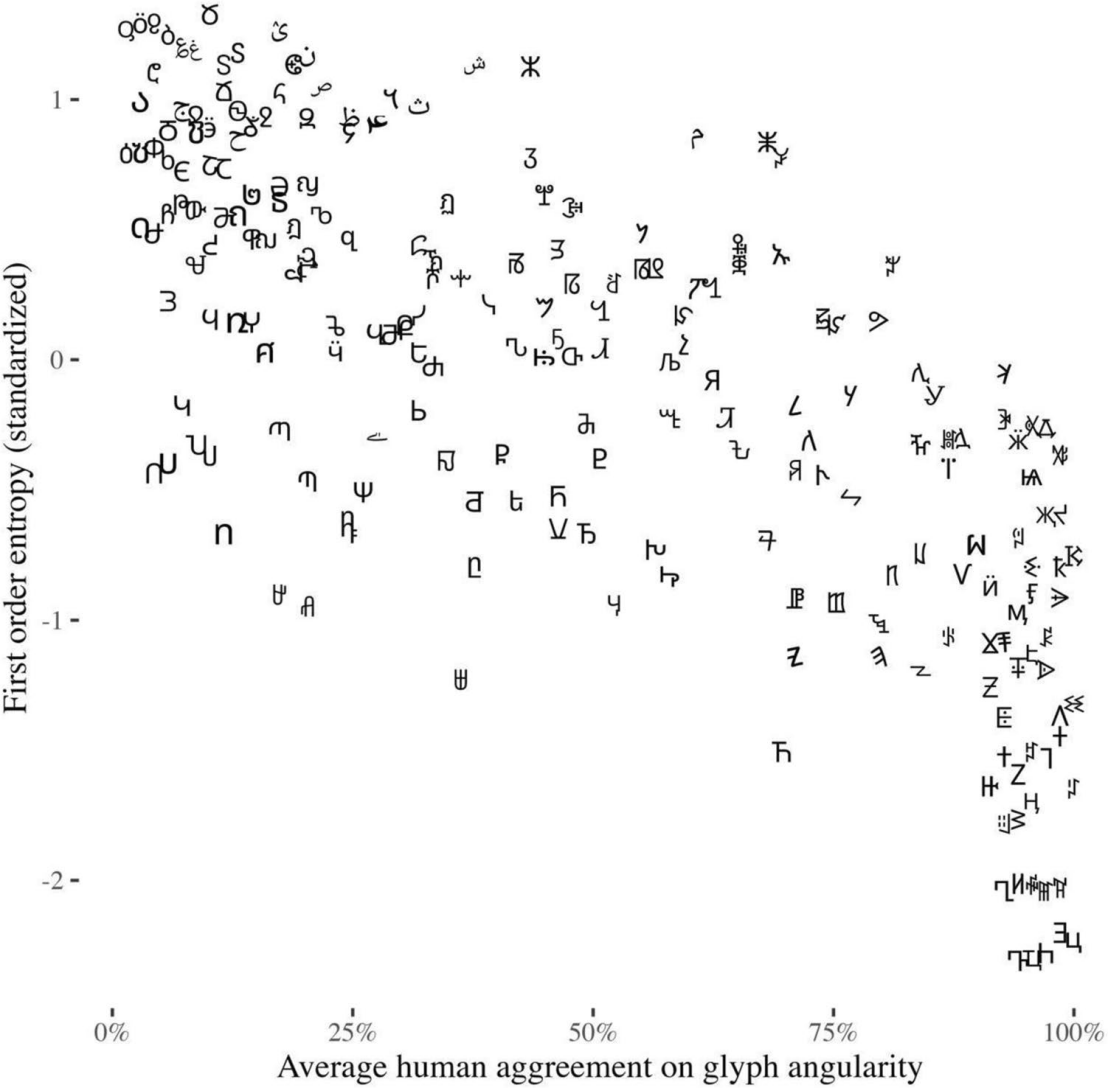
Average human agreement on glyph angularity plotted against first-order Shannon entropy

For human data, we report simple percentages of judgments of angular shape. In the dataset, the variable <avg_response> is the mean of angularity for each glyph, where 1 is angular and 0 is round (i.e., a value of 1 shows that every subject chose angular for a particular glyph; Fig. 1).

We then validated the metrics using the human measure of angularity, by means of Pearson’s correlation coefficients between each metric and the <avg_response> variable. We found that first-order entropy of edge orientation has the strongest association with human-judged angularity as compared with the other angularity metrics (*r =* –.84, 95% CI [–.87, –.81], *p* <.001). This accords with Watier’s (2024) result, as reported in Table 9 in the original study. All other correlations between computational metrics and human judgment are presented in Table 1. Knowing that first-order entropy of edge orientation is strongly associated with human judgment in shape angularity classification tasks allows its use to norm new glyphs (or other visual stimuli) without needing to go through another full norming study.

**Table 1:** Descriptive statistics and correlations

Variable	*N*	*M*	*SD*	1	2	3	4	5
1. avg_response	400	0.44	0.37					
2. peaks_s_theta	400	1.04	0.78	.57**				
				[.50,.63]				
				*p* <.001				
3. first_order	400	3.67	0.67	-.84**	-.43**			
				[-.87, -.81]	[-.51, -.35]			
				*p* <.001	*p* <.001			
4. second_order	400	2.45	0.51	-.26**	-.03	.55**		
				[-.35, -.17]	[-.13,.07]	[.47,.61]		
				*p* <.001	*p* =.566	*p* <.001		
5. CPDA_maxima	400	106.57	16.92	.54**	.39**	-.38**	.19**	
				[.46,.60]	[.30,.47]	[-.46, -.30]	[.09,.28]	
				*p* <.001	*p* <.001	*p* <.001	*p* <.001	
6. scalar_q_maxima	400	12.31	8.62	.66**	.14**	-.76**	-.43**	.39**
				[.60,.71]	[.05,.24]	[-.80, -.72]	[-.50, -.34]	[.31,.47]
				*p* <.001	*p* =.004	*p* <.001	*p* <.001	*p* <.001

In addition to computational and human measures of shape features, we also provide additional contextual details about each of the glyphs in order to make the data relevant to ongoing work in cognitive linguistics and anthropology. Those variables include hexadecimal Unicode codes (<unicode_number>), character representations of the Unicode string (<character>), script (<script>), transliteration (<transliteration>; automated with AnyAscii), and description (<glyph_description>). Contextual details were manually collected from Wiktionary (https://www.wiktionary.org/), PHOIBLE 2.0 (http://phoible.org; Moran & McCloy, 2019), and the Unicode Character Database (https://www.unicode.org/ucd/).

## Discussion

Our intention has been to create a psycholinguistic database of normed orthographic stimuli of use to a broad scientific community. We have attempted to show that, despite the linguistic, evolutionary, and graphical complexity of writing systems, measures of visual dimensions such as angularity can be extracted from orthographic stimuli to be used in both behavioral and computational research programs. Moreover, we have provided a replicable, relatively accessible use case of recent breakthroughs in computational analysis of visual features and have shown how behavioral researchers can leverage powerful computing methods to rigorously generate metrics for stimuli sets. For example, we have used this normed set of stimuli set to generate the materials for a behavioral experiment testing the sound symbolic features of various writing systems (Porto et al., 2024).

The present norms have broad applications in psycholinguistics, cognitive anthropology, cognitive neuroscience, and experimental psychology. For example, researchers studying attention, perception, and learning could use the stimuli set to test how glyph shape affects recognition speed and accuracy across different writing systems, in a way similar to Preziosi and Coane (2017). Researchers could investigate the effects of character shape on memory retention and learning efficiency, in both first- and second-language acquisition, or whether accentuating the shape features of characters aids in learning for persons with reading disabilities. For example, sound symbolic features of writing have been shown to help adults and children with word learning and predict reading outcomes longitudinally (Horbach et al., 2018; Imai & Akita, 2024). Functional magnetic resonance imaging (fMRI) and event-related potential (ERP) studies could utilize these stimuli to explore the temporal dynamics associated with visual processing of round and angular characters; the stimuli could be paired with audio (e.g., phonetic pronunciation) to see if and how the brain integrates visual and auditory information across different shape and sound features. Cognitive anthropologists could use these stimuli to study how the physical properties of characters have evolved in different writing systems in response to different linguistic and cultural factors. We hope that this new stimuli set encourages future research aimed at investigating the neurocognitive, behavioral, and evolutionary relationship between written and spoken language.

## Conclusion

This manuscript presents a database of 3,208 glyphs normed on shape angularity by means of computational methods. A subset of 400 glyphs were also normed by asking participants to classify the glyphs as either angular or curvilinear. The human judgments were used to validate the computational measures. All computational measures, but in particular first-order entropy of edge orientation, correlated highly with human angularity judgments. We also described how this measure can be obtained for new glyphs or any other kinds of shapes. We hope that these tools will be of help to researchers investigating the complex relationship between human writing and speech.

## Supplementary information

Below is the link to the electronic supplementary material.

Supplementary file1 (XLSX 354 KB)

## Data Availability

Data and MATLAB code can be found at the following link: https://osf.io/h2kdq/

## Footnotes

1 Readers interested in replicating the glyph generation code must install the relevant Noto typefaces for different scripts prior to running the code. See: https://fonts.google.com/noto for a subset of them, a majority of which are logograms.

2 The only dependency that must be installed outside of MATLAB is the Digital Image Processing Using MATLAB Toolbox 3, which can be found with installation instructions here: https://github.com/dipum/dipum-toolbox
